# Adherence to Mediterranean Healthy Lifestyle Patterns and Potential Barriers: A Comparative Study of Dietary Habits, Physical Activity, and Social Participation Between German and Turkish Populations

**DOI:** 10.3390/nu17213338

**Published:** 2025-10-23

**Authors:** Achraf Ammar, Ayse Merve Uyar, Atef Salem, Ludwig Álvarez-Córdova, Mohamed Ali Boujelbane, Khaled Trabelsi, Bekir Erhan Orhan, Juliane Heydenreich, Christiana Schallhorn, Giuseppe Grosso, Evelyn Frias-Toral, Haitham Jahrami, Piotr Zmijewski, Hamdi Chtourou, Wolfgang I. Schöllhorn

**Affiliations:** 1 Department of Training and Movement Science, Institute of Sport Science, Johannes Gutenberg-University Mainz, 55122 Mainz, Germany; auyar@students.uni-mainz.de (A.M.U.); asalem@uni-mainz.de (A.S.); mboujelb@uni-mainz.de (M.A.B.); wolfgang.schoellhorn@uni-mainz.de (W.I.S.); 2 High Institute of Sport and Physical Education of Sfax, University of Sfax, Sfax 3000, Tunisia; khaled.trabelsi@isseps.usf.tn (K.T.); hamdi.chtourou@isseps.usf.tn (H.C.); 3 Research Laboratory, Molecular Bases of Human Pathology, LR19ES13, Faculty of Medicine of Sfax, University of Sfax, Sfax 3000, Tunisia; 4 Carrera de Nutrición y Dietética, Facultad de Ciencias de la Salud, Universidad Católica de Santiago de Guayaquil, Guayaquil 090615, Ecuador; ludwig.alvarez@cu.ucsg.edu.ec; 5 Maestría de Nutrición y Dietética, Facultad de Ciencias de la Salud, Universidad de Las Américas (UDLA), Quito 170124, Ecuador; 6 Department of Movement Sciences and Sports Training, School of Sport Science, The University of Jordan, Amman 11942, Jordan; 7 Research Laboratory: Education, Motricity, Sport and Health, EM2S, LR19JS01, High Institute of Sport and Physical Education of Sfax, University of Sfax, Sfax 3000, Tunisia; 8 Faculty of Sports Sciences, Istanbul Aydın University, 34295 Istanbul, Türkiye; bekirerhanorhan@aydin.edu.tr; 9 Department of Experimental Sports Nutrition, Faculty of Sports Sciences, Leipzig University, 04109 Leipzig, Germany; 10 Department of Sports Economics, Sociology and History, Institute of Sport Science, Johannes Gutenberg-University Mainz, 55122 Mainz, Germany; christiana.schallhorn@uni-mainz.de; 11 Department of Biomedical and Biotechnological Sciences, University of Catania, 95124 Catania, Italy; 12 Escuela de Medicina, Universidad Espíritu Santo, Samborondón 0901952, Ecuador; 13 Division of Research, Texas State University, 601 University Dr, San Marcos, TX 78666, USA; 14 Government Hospitals, Manama 973, Bahrain; haitham.jahrami@outlook.com; 15 Department of Psychiatry, College of Medicine and Medical Sciences, Arabian Gulf University, Manama 329, Bahrain; 16 Department of Biochemistry, Gdansk University of Physical Education and Sport, 80-336 Gdansk, Poland; piotr.zmijewski@insp.pl

**Keywords:** Mediterranean diet, physical activity, social participation, Germany, Türkiye, lifestyle barriers, cultural influence, dietary awareness

## Abstract

**Background/Objectives**: Adherence to the Mediterranean Diet (MedDiet) has declined even within Mediterranean regions, while its adoption has become more common in non-Mediterranean contexts. This study compares Germany and Türkiye, two culturally contrasting contexts traditionally classified as non-Mediterranean and Mediterranean, respectively, to examine cross-cultural differences and patterns in MedDiet adherence and related lifestyle behaviors. Specifically, it aims to compare adherence to the Mediterranean lifestyle (MedLife), physical activity, and social participation, and to analyze their associations within each country. **Methods**: Using data from the MEDIET4ALL survey, 1184 valid responses (609 from Germany and 575 from Türkiye) were analyzed for dietary behaviors, perceived barriers to MedDiet adherence, physical activity, and social engagement, with adherence assessed via the MEDLIFE index. **Results**: The majority of respondents were healthy (79%), employed (67%), young adults (56%), of normal weight (51%), living in urban environments (72%), and showed a gender balance (52.5% female). Most were classified as medium MedDiet adherent in both Germany (45%) and Türkiye (56%), with no significant difference in total MedLife scores. However, block- and item-level analyses revealed that Turkish participants showed higher adherence to Mediterranean food consumption (*p* < 0.001), particularly in limiting processed meat and consuming legumes, dairy, nuts/olives, and olive oil. In contrast, German participants adhered more closely to recommendations for red meat and cereal intake. German participants also scored higher on lifestyle-related behaviors (e.g., regular napping and recommended sleep duration), while no significant differences were found in the dietary habits block. Awareness of the MedDiet was significantly higher among German participants (*p* < 0.001), with country-specific differences in perceived barriers (i.e., higher total score among Germans with *p* = 0.03). Germans reported more barriers related to social norms and health conditions, while Turkish respondents more often cited attitudes, cost, and individual beliefs. Physical activity levels were significantly higher in Germany, whereas Turkish respondents reported greater social participation (*p* < 0.001). Weak to moderate correlations (r = 0.09 to 0.035) were found between MedLife adherence and both physical activity and social participation, with stronger associations observed among German participants. **Conclusions**: These findings highlight the culturally embedded nature of lifestyle behaviors related to MedDiet adherence. Despite similar overall adherence levels, Germans and Turks differ in specific dietary patterns, lifestyle practices, and perceived barriers, underscoring the need for culturally tailored interventions to improve adherence.

## 1. Introduction

Health behaviors such as physical activity, nutrition, and social engagement are fundamental determinants of human well-being [1]. Among various dietary patterns, the Mediterranean diet (MedDiet) is widely recognized as the world’s most evidence-based eating pattern for promoting health and longevity while contributing to environmental sustainability by conserving water and energy, reducing greenhouse gas emissions, and preserving land resources [2,3,4]. Notably, expert consensus has identified the MedDiet as one of the easiest healthy eating patterns to follow, due to its biodiversity, strong sociocultural roots, and positive economic impact at the local level [3,5,6]. Beyond its food components, the modern MedDiet pyramid integrates elements of an active lifestyle, as well as psychosocial and cultural traditions, such as sharing meals with family [7], which has led to a broader conceptualization of the Mediterranean lifestyle (MedLife) in recent research [6,8,9,10].

The MedDiet emphasizes primarily plant-based, minimally processed foods, complemented by moderate consumption of dairy, fish, poultry, and red wine, aligning with sustainable agricultural and environmental practices [11,12,13,14].

Over the years, extensive research has highlighted the MedDiet’s role in preventing cardiovascular diseases (CVDs), obesity, type 2 diabetes, and neurodegenerative disorders [15,16,17]. Large cohort and interventional studies, including the Seven Countries Study and PREDIMED trial, have consistently shown strong cardiometabolic benefits [18,19,20].

Despite its well-established health and sustainability benefits, adherence to the MedDiet has been steadily declining in recent decades, even within Mediterranean countries [8]. This trend is largely driven by the increasing consumption of ultra-processed foods (UPFs) and the gradual shift toward Westernized dietary and lifestyle patterns, which are displacing traditional eating habits and cultural practices [21]. Studies on adolescents and older adults in Türkiye have shown poor compliance, with many individuals failing to meet recommended dietary guidelines [22,23].

The factors influencing MedDiet adherence are multifaceted, encompassing socioeconomic status, cultural norms, food accessibility, and education levels [24,25]. Furthermore, there is a growing body of evidence suggesting that social participation and physical activity play a crucial role in maintaining dietary habits [10]. Social interactions provide motivation and support for healthy eating behaviors, while an active lifestyle complements the dietary benefits of the MedDiet [26,27]. However, limited research has explored the interplay between dietary adherence, physical activity, and social participation in diverse populations.

The MedDiet appears to vary across Mediterranean and non-Mediterranean countries [8], particularly in the context of increasing globalization and migration. Germany and Türkiye present an interesting case for comparison, as they have distinct cultural and dietary backgrounds but share significant social and economic ties. While Türkiye is geographically located in the Mediterranean region, recent studies suggest that adherence to the MedDiet is not as strong as expected [28]. In contrast, Germany, despite not being a Mediterranean country, has seen a rising trend in MedDiet adoption due to increased health awareness and food accessibility [29].

However, most existing studies have investigated dietary patterns in either Germany or Türkiye independently, often using different assessment tools or questionnaires, making direct comparison challenging. There is a notable lack of comprehensive cross-country studies using standardized methodologies to jointly evaluate adherence to the MedDiet and associated lifestyle factors.

To address this gap, the present study is, to our knowledge, the first to apply a harmonized and validated set of assessment tools to directly compare Mediterranean lifestyle adherence and related behaviors between Türkiye and Germany, two culturally contrasting countries representing Mediterranean and non-Mediterranean contexts.

As part of the wider MEDIET4ALL PRIMA project [6], the present study aims to compare MedDiet adherence and barriers, physical activity levels, and social participation between German and Turkish populations and to examine the associations among these lifestyle components within each country. This approach allows the identification of cross-cultural patterns that may reflect contextual differences in lifestyle behaviors and is intended to inform recommendations for promoting adherence to MedLife. It is hypothesized that, due to cultural and lifestyle differences, Turkish participants would report higher adherence to the MedDiet and greater social participation, whereas German participants would demonstrate higher levels of physical activity.

## 2. Materials and Methods

### 2.1. Promotion and Development of Surveys

In this cross-sectional study, an international online survey conducted in multiple languages, referred to as the MEDIET4ALL survey [8,9,10], was used to examine adherence to the MedDiet and associated lifestyle behaviors. The survey was designed, reviewed, and edited by a multidisciplinary team of researchers, including public health, nutrition, sports and movement sciences, psychology, and sociology experts involved in the MEDIET4ALL PRIMA project, supported by the European Union [8,9,10]. The survey underwent a one-week pilot test conducted by the project’s steering group prior to its dissemination. Based on the feedback received, the survey was finalized and subsequently launched across ten Mediterranean and neighboring countries (Germany, France, Italy, Spain, Luxembourg, Tunisia, Algeria, Morocco, Türkiye, and Jordan) over a four-month period in the summer of 2024. The survey was administered and promoted by organizations from Europe, North Africa and Western Asia. To increase the accessibility of the survey, it was translated into seven languages: English, German, French, Italian, Spanish, Arabic and Turkish. Items without official translations were translated and back-translated very strictly, and the test–retest reliability coefficients were good to excellent (r = 0.81–0.94) for all translated items. The survey consisted of 75 items, most of which were adopted from established questionnaires to measure MedLife adherence, challenges, and determinants of geo-demographic and socioeconomic characteristics, health status, mental health, life satisfaction, and multidimensional lifestyle behaviors (for example, physical activity and social participation). The survey was conducted using the SoSci Survey tool, which is a General Data Protection Regulation (GDPR)-compliant web application hosted by Johannes Gutenberg University infrastructure. The link to the survey was sent by the MEDIET4ALL consortium and collaborators (e.g., Bilendi Solution) through different channels, which included email invitations, official university and consortium websites and pages, the MEDIET4ALL study website, and social media platforms including ResearchGate™, LinkedIn™, Facebook™, WhatsApp™ and X™ (formerly Twitter). Furthermore, people were encouraged to share the survey link with their friends and families. Notably, for the purpose of the present paper, it is important to highlight that dissemination efforts were primarily concentrated in Istanbul, whereas in Germany, the campaign was conducted across multiple cities to ensure broader geographic representation.

The first page of the survey introduced the study, the rationale, the goals, the ethical considerations, the data collection and privacy issues, and the consent form, followed by a language selection menu offering the seven available linguistic versions. The survey did not collect any personally identifiable information, such as names or contact details, from the participants. Respondents could exit the survey at any time without completing it; in such cases, their data were not saved. Only responses submitted via the final “Submit” button were stored.

To ensure data quality, several validation measures were implemented. Logical filters and consistency checks were used to identify incomplete or contradictory responses, for example, indicating no engagement in vigorous physical activity while simultaneously reporting daily participation in such activity. Duplicate responses were identified and excluded based on IP address, time stamps, and demographic/survey data. Additional data cleaning steps included excluding responses with implausible or extreme values, such as reporting 24 h of sleep [8,9,10]. The sampling strategy was designed to prioritize broad population representativeness, aiming to capture diverse demographic profiles. Inclusion criteria required participants to be aged 18 years or older and to have provided informed consent. Only respondents who completed all survey questionnaires were included in the analysis. Exclusion criteria encompassed individuals who self-reported cognitive or memory-related impairments (e.g., cognitive decline, mild cognitive impairment, or dementia), as such conditions could compromise the reliability of self-reported data. The study was conducted in accordance with the ethical principles outlined in the Declaration of Helsinki. The research protocol, including informed consent procedures, received formal approval from the Ethics Committee.

### 2.2. Sample Size

The study employed a broad approach in selecting the sample to enhance participant diversity and statistical power. While the sample size was not explicitly calculated, the study aimed to gather data from a large number of participants. The initial sample consisted of over 8000 participants from various regions, including Türkiye and Germany. A total of 4010 responses were identified as valid and complete, while the remaining responses (n = 3990) were excluded due to age or consent issues, self-reported cognitive impairments, incomplete surveys, logical inconsistencies, duplicate entries, or implausible values, as detailed in the previous section. A subset of 1184 valid responses from Türkiye (n = 575) and Germany (n = 609) was analyzed in this study. A post hoc power analysis was conducted using G*Power 3.1 to determine whether the final sample size (N = 1184; Germany = 609, Türkiye = 575) provided sufficient statistical power. Assuming a two-tailed independent samples *t*-test, an alpha level of 0.05, and an effect size of d = 0.26, corresponding to the observed difference in Block 1 of the MedLife Index (Mediterranean Food Consumption Patterns), which is the most weighted component based on the number of items included. The resulting statistical power (1-β) was 0.99, indicating that the sample size was sufficient to detect small to moderate group differences and ensure the reliable detection of meaningful effects.

### 2.3. Data Privacy

The inclusion into the study was voluntary, and there was no need to justify the refusal to participate, withdraw from the study or terminate participation at any time. To guarantee ethical compliance, participants were informed that (i) all data collected would be used for research purposes only, and (ii) responses would be anonymous and confidential as stated in the SoSci Survey privacy policy (www.soscisurvey.de/en/privacy, accessed on 1 July 2024). The survey was compatible with the Federal Data Protection Act (BDSG) and the EU General Data Protection Regulation (GDPR). Thus, when the questionnaire was completed and submitted, the participants’ consent to use their anonymous data for research purposes was assumed.

### 2.4. Survey Questionnaires

The survey incorporated a range of validated questionnaires along with targeted items to comprehensively assess adherence to MedLife and its associated factors. In the present study, the primary focus is to evaluate participants’ adherence to MedLife and their engagement in physical and social activities, as well as to collect various demographic and socioeconomic characteristics.

#### 2.4.1. MedLife Index

The adherence scores to MedDiet were recently assessed in a systematic review carried out by Zaragoza-Martí et al. [30]. Of the 28 scores analyzed, 12 were applied to general populations, with only five concerning MedDiet Pyramid principles [7,31]. However, the “MEDLIFE Index,” developed by Sotos-Prieto et al. [32], showed good internal consistency (Cronbach’s α = 0.75). More recently, the scale was translated and validated in Turkish adults: a 2024 adaptation study involving 300 participants confirmed its construct validity and demonstrated strong test–retest reliability (ICC = 0.82), providing the first cross-cultural evidence for the MEDLIFE Index [33]. Based on these findings, the MEDLIFE Index was selected for inclusion in the MEDIET4ALL e-survey.

The MEDLIFE Index, a validated instrument, evaluates adherence to MedDiet principles and related lifestyle factors [32]. Structured around the MedDiet Pyramid [31], it includes 28 items classified into three categories: food consumption frequency (15 items), dietary habits (7 items), and lifestyle behaviors (6 items). The first category assesses consumption frequency of key MedDiet components, such as fruits, vegetables, whole grains, and healthy fats, while monitoring reduced intake of pastries and red meat. The second category evaluates habits like minimizing added salt/sugar and avoiding between-meal snacking. The third category quantifies lifestyle behaviors, measuring physical activity (≥150 min of moderate intensity), sleep duration (6–8 h), and social engagement.

Items are scored 0 (non-adherence) or 1 (adherence), with total scores ranging from 0 (lowest) to 28 (highest). Adherence levels are categorized as low (<12), medium (12–16), or high (>16), based on tertile distributions. This tiered scoring provides a holistic evaluation of MedDiet and lifestyle adherence.

#### 2.4.2. International Physical Activity Questionnaire Short Form (IPAQ-SF)

The IPAQ-SF is a self-report questionnaire measuring physical activity levels (vigorous, moderate, and walking) over a week, expressed as MET-minutes/week. Activity is categorized as low (<1500), moderate (1500–2999), or high (≥3000). Widely used in research and clinical settings, it evaluates activity patterns in diverse populations [34,35]. The IPAQ-SF was validated in the Turkish and German languages: for the Turkish version, the test–retest reliability Spearman correlation coefficient was r = 0.69 [36], while for the German version, the reliability was r = 0.43–0.68 and the validity r = 0.39 [37].

#### 2.4.3. Short Social Participation Questionnaire (SSPQ-L)

The SSPQ-L, a brief adaptation of the Social Participation Questionnaire (SPQ, acceptable internal reliability, with PSI: 0.71–0.74), measures social participation over the past year and was validated during COVID-19 home confinement [38,39]. It comprises 14 items: 10 use a 5-point scale (“never” to “all the time”), and 4 are yes/no. Total scores (14–70) reflect participation levels, categorized as 14 (“never socially active”), 15–28 (“rarely”), 29–42 (“sometimes”), 43–56 (“often”), and 57–70 (“socially active at all times”).

#### 2.4.4. The MedDiet Barriers Questionnaire (MBQ)

The MedDiet Barriers Questionnaire (MBQ) is a novel 13-item questionnaire developed through a multi-stage process within the broader framework of the MEDIET4ALL EU project to assess perceived barriers and limitations to MedDiet adherence [8,10]. Participants respond “Yes” or “No” to barriers spanning domains such as food allergies/intolerances, cultural/religious restrictions, medical limitations, personal dietary choices (e.g., vegan/vegetarian diets), taste preferences, attitudinal concerns (e.g., perceived suitability, food waste), social norms, low motivation, financial constraints, time-intensive meal preparation, limited food accessibility, lack of culinary skills, and other factors. Each “Yes” (barrier present) scores 1 point, yielding a total score of 0–13. Higher scores indicate greater adherence challenges, with 0 reflecting no barriers and 13 representing severe limitations.

#### 2.4.5. Additional Questions

Additional questions captured geo-demographic, socioeconomic, and health-related characteristics, as well as participants’ awareness of the MedDiet. Variables included age, gender, marital status, education level, employment status, living environment, country of residence, ethnicity, smoking habits, and body mass index (BMI). BMI was estimated based on self-reported weight and height provided by the participants. The BMI was calculated using the standard formula recommended by the World Health Organization (WHO): BMI = weight (kg)/[height (m)]^2^. Based on this calculation, participants were classified into four categories using WHO thresholds: underweight (<18.5 kg/m^2^), normal weight (18.5–24.9 kg/m^2^), overweight (25.0–29.9 kg/m^2^), and obese (≥30.0 kg/m^2^). Self-reported health status was categorized as healthy, at risk of disease, or living with disease. The “at risk” category was defined by the presence of two or more cardiovascular risk factors, a history of cardiovascular disease, diabetes mellitus, or a combination of these conditions, as previously detailed by Ammar et al. [10]. Information on participants’ exact cities of residence was not collected, as the study focused on classifying living environment (urban, suburban, and rural), which could better capture the contextual influences on dietary behavior, accessibility, and lifestyle patterns across regions.

### 2.5. Statistical Analysis

The data was analyzed by IBM SPSS (Statistical Package for Social Sciences) software version 29.0. Descriptive statistics were presented as means and standard deviations since the normality of the distribution of the data was confirmed by checking skewness and kurtosis values in addition to histograms. Therefore, parametric tests were preferred instead of their non-parametric counterparts. The chi-square test was used to investigate the relationship between two categorical variables. An independent sample *t*-test was used to compare two independent groups. Pearson correlation analysis was utilized to check the relationship between two variables with continuous score values. Pearson correlation analysis was used to assess the relationship between continuous variables. Correlation strength was interpreted based on commonly accepted thresholds: r < 0.10 was considered negligible, 0.10 ≤ r < 0.30 weak, 0.30 ≤ r < 0.50 moderate, and r ≥ 0.50 strong [40]. In all statistical tests, the significance level was set as *p* < 0.05.

## 3. Results

### 3.1. Demographic Characteristics of the Responders 

Demographic characteristics of participants are summarized in Table 1. Cross-country differences were significant between Türkiye and Germany in all tested variables except gender and health status (*p* = 0.046 for BMI and <0.0001 for the remaining variables). Türkiye presented a higher proportion of people living in urban areas (standardized residual = 4.6, *p* < 0.001), young adults (residual = 2.2, *p* < 0.001), and underweight and overweight cases (residual = 1.9 and 1, *p* < 0.05). Germany reported a higher proportion of people in suburban and rural environments (residuals = 4.8 and 5.3, respectively; *p* < 0.001), older adults (residual = 6.0, *p* < 0.001), and obesity cases (residuals = 1.8 and 1.1; *p* < 0.05). Additionally, Türkiye reported higher rates of no formal schooling and bachelor’s degrees (residual = 5.2 and 5.0; *p* < 0.001, respectively) and married status (residual = 3.1, *p* < 0.001), whereas Germany presented higher proportion of people with professional degrees and advanced qualifications (residual = 7.1, *p* < 0.001) (master’s/doctoral degree: residual = 7.1 and 2.6, *p* < 0.01 and 0.001, respectively) and single (residual = 1.6, *p* < 0.05) and widowed/divorced individuals (residual = 1.6 and 3.3, *p* < 0.001 and 0.05, respectively). Moreover, Türkiye had a significantly higher proportion of unemployment (residual = 3.3, *p* < 0.001), higher proportion of daily shisha use (residual = 4.9), and lower proportion of non-smokers (residual = −3.4, *p* < 0.001) versus higher retirement rates in Germany (residual = 3.4, *p* < 0.001) and lower proportion of daily shisha use and higher proportion of non-smokers (residual = −4.8 and 3.3; all *p* < 0.001).

### 3.2. Medlife Index

**Figure 1:**
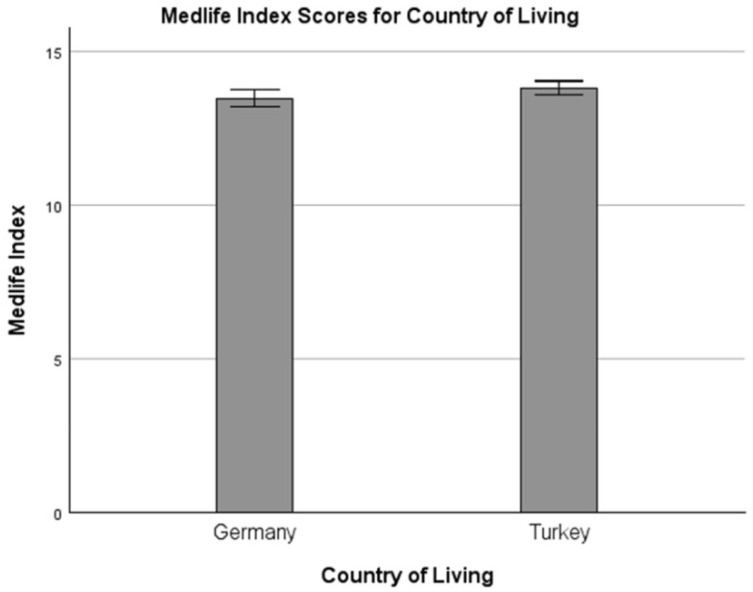
Total score of MedLife Index among German vs. Turkish responders.

The total scores for the MedLife index among German vs. Turkish participants are presented in Figure 1. The scores for Block 1, “Mediterranean Food Consumption,” Block 2, “Mediterranean Dietary Habits,” and Block 3, “Activity,” of the Medlife Index, as well as the participants’ classification into low, moderately and highly adherent to MedLife, are presented in Table 2.

#### 3.2.1. Block 1: Mediterranean Food Consumption Patterns

Turkish participants scored significantly higher than German participants in Mediterranean Food Consumption (t(1175.6) = −4.53, *p* < 0.001, d = −0.26), as reflected by higher mean scores in Block 1 of the MedLife index (Türkiye: 6.87 ± 1.73 vs. Germany: 6.38 ± 1.98). Specifically, Turkish respondents demonstrated greater adherence to recommended serving frequencies for processed meat, legumes, dairy, nuts/olives, white meat, and olive oil (*p* < 0.001). Conversely, German participants showed higher adherence to MedDiet recommendations for red meat, eggs, fish, and cereal consumption. No significant differences (*p* > 0.05) were found for the remaining five items (sweets, potatoes, herbs/spices, fruits, or vegetables).

#### 3.2.2. Block 2: Mediterranean Dietary Habits

No significant difference in mean scores was observed for Mediterranean Dietary Habits (t(1182) = −0.21, *p* = 0.835, d = −0.01; Germany: 4.13 ± 1.68 vs. Türkiye: 4.15 ± 1.57). However, item-level analysis within Block 2 revealed that Turkish participants showed greater adherence to MedDiet recommendations regarding salt intake, grain products, snacks, and processed foods (*p* < 0.05). Conversely, German participants adhered more closely to the recommended intake of wine (χ^2^ = 18.00, *p* = 0.001) and sugar in beverages. No statistically significant differences were found between the two groups in terms of overall drink consumption and limiting nibbling between meals.

#### 3.2.3. Block 3: Physical Activity, Rest, Social Habits, and Conviviality

German participants had significantly higher mean scores than Turkish participants in Block 3: “Physical activity, rest, social habits, and conviviality” (t(1182) = 2.22, *p* = 0.027, d = 0.13; Germany: 2.99 ± 1.27 vs. Türkiye: 2.82 ± 1.30). Germans reported higher adherence to recommended physical activity levels, regular napping, and 6–8 h of sleep (*p* < 0.05). In contrast, a greater proportion of Turkish participants reported limiting TV time to less than 1 h per day (*p* = 0.001). No significant country-specific differences were found for spending time with friends or participation in team sports.

#### 3.2.4. MedLife Index’s Total Score and Category Classifications

Most participants exhibited medium adherence (~50%) to MedLife, with no statistically significant difference in total MedLife scores between Germany (13.49 ± 3.41) and Türkiye (13.79 ± 2.76) (t(1152.77) = −1.90, *p* = 0.058, d = −0.11) (Figure 1). However, the distribution of adherence categories differed significantly between the two countries (χ^2^ = 10.579, *p* = 0.005). Türkiye had a significantly higher proportion of participants classified in the medium adherence category (standardized residual = 2.4, *p* < 0.05), whereas Germany had significantly higher proportions of participants classified in the low adherence (standardized residual = 2.2, *p* < 0.05) and high adherence (standardized residual = 2.0, *p* < 0.05) categories compared to Türkiye.

### 3.3. MedDiet Awareness and MedDiet Barriers Questionnaire (MBQ) Scores

Table 3 presents the participants’ responses regarding awareness of the MedDiet and perceived barriers to its adoption, as assessed by the MBQ. Awareness of the MedDiet was significantly higher among German respondents (55.7%) compared to Turkish participants (38.3%; χ^2^ = 35.95, *p* < 0.001). Significant cross-country differences were also observed across multiple perceived barriers. Turkish respondents reported significantly higher proportions of perceived barriers related to attitudes (*p* < 0.05), price affordability, and individual beliefs (*p* < 0.001). In contrast, German respondents reported higher perceived barriers related to social norms, time/effort for consuming (*p* < 0.01), lack of knowledge/cooking skills (*p* < 0.01), food allergies and intolerances, cultural and/or religious reasons (*p* < 0.001), and medical reasons (*p* < 0.05). No significant differences were observed for low motivation, low accessibility/availability of Mediterranean food, taste dislike or the “other” category. Finally, a comparison of the MBQ total score revealed that German participants had significantly higher overall perceived barrier scores (7.09 ± 1.24) compared to Turkish participants (6.94 ± 1.21), Z = −2.16 and *p* = 0.031.

### 3.4. Physical Activity and Social Participation

Table 4 summarizes the classification of participants into different physical activity and social participation levels based on their total scores from the IPAQ-SF and SSPQ-L questionnaires. Overall, the majority of participants were categorized as low physically active (57%) and sometimes socially active (50%). However, the distribution of these classifications differed significantly between countries (*p* < 0.001 for IPAQ-SF; *p* = 0.006 for SSPQ-L). A higher proportion of Turkish participants were classified as having low physical activity levels (standardized residual = 4.2, *p* < 0.001), whereas German participants showed significantly higher proportions in both the moderately active (residual = 2.6, *p* < 0.01) and highly active (residual = 3.9, *p* < 0.001) categories. Regarding the SSPQ-L, Türkiye had a significantly higher proportion of participants in the often socially active category (residual = 2.6, *p* < 0.01), while no significant differences were observed between countries for the other social participation categories.

**Figure 2:**
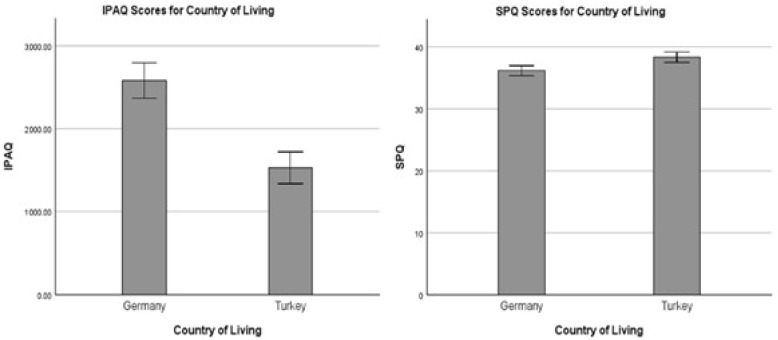
Total score of IPAQ-SF and SSPQ-L among German vs. Turkish responders.

In terms of total scores, the country of living significantly affected both IPAQ-SF and SSPQ-L total scores (Figure 2). German participants reported significantly higher physical activity levels compared to Turkish participants (t(1182) = 7.158, *p* < 0.001), with mean values of 2563.02 ± 2674.81 and 1513.62 ± 2320.56 MET-minutes/week for Germany and Türkiye, respectively. Conversely, Turkish respondents showed notably higher SSPQ-L total scores compared to German (t(1152) = −3.617, *p* < 0.001), with mean values of 38.52 ± 10.54 and 36.25 ± 10.15 for Türkiye and Germany, respectively.

### 3.5. Correlation Between Total Scores of MedLife Index, IPAQ-SF, and SSPQ-L

Table 5 presents the correlation scores between total scores of the MedLife Index, IPAQ-SF, and SSPQ-L total scores for both German and Turkish respondents. Among German participants, MedLife scores showed a weak but significant correlation with IPAQ-SF (r = 0.092, *p* = 0.023) and a moderate positive correlation with SSPQ-L (r = 0.349, *p* < 0.001). Additionally, IPAQ-SF and SSPQ-L were weakly correlated (r = 0.159, *p* < 0.001). Among Turkish respondents, there was no significant correlation between MedLife and IPAQ-SF scores (r = 0.071, *p* = 0.090). However, MedLife scores showed a weak but significant correlation with SSPQ-L (r = 0.093, *p* = 0.029). A weak but significant correlation was also observed between IPAQ-SF and SSPQ-L scores (r = 0.178, *p* < 0.001).

## 4. Discussion

This study compared German and Turkish populations in terms of their adherence to the MedDiet, perceived barriers, physical activity levels, and social participation, using the MedLife Index, IPAQ-SF, and SSPQ-L. Overall, the findings provide valuable insights into how sociodemographic and cultural factors may shape lifestyle behaviors in different contexts. Although no significant differences were observed in total MedLife adherence scores, country-specific patterns emerged. Turkish participants showed greater adherence to Mediterranean food consumption practices, while German participants scored higher in lifestyle-related behaviors, particularly regarding physical activity, regular rest, and sleep. Awareness of the MedDiet was also higher among German respondents, who simultaneously reported more cultural and medical barriers. In contrast, Turkish participants more frequently cited attitudinal and financial obstacles. Moreover, higher social participation was linked with greater MedLife adherence in both countries, although physical activity was only associated with MedLife scores in the German sample. These findings highlight that cultural and environmental contexts distinctly shape how populations integrate the components of the MedLife. While the Turkish sample was generally younger and more urban, and the German sample was older with higher formal education, causality cannot be inferred due to the cross-sectional design.

### 4.1. Demographic and Socioeconomic Differences

The demographic and socioeconomic disparities between Türkiye and Germany were reflected in the study’s findings. Türkiye’s higher urbanization rate among participants aligns with national statistics [41] and is also likely influenced by the concentration of data collection efforts in major metropolitan areas such as Istanbul. Furthermore, the younger age profile of Turkish respondents mirrors the broader demographic structure of the country, where the median age is significantly lower than that of Germany [42,43].

Although there were no significant differences in overall BMI distributions between the two samples, the underlying patterns varied. Türkiye had a greater proportion of both underweight and overweight individuals, while Germany reported slightly higher rates of obesity. These trends are consistent with existing literature, which indicates rising obesity prevalence in both countries, with higher obesity rates among German men [44] and a growing pre-obesity trend among Turkish men [45].

Education levels also differed significantly, with Türkiye having a larger proportion of individuals who had not completed formal schooling. This is consistent with OECD data, which indicates that 44% of Turkish adults lack upper secondary education, compared to only 13% in Germany [46]. Interestingly, Türkiye had a higher proportion of bachelor’s degree holders, likely reflecting the country’s education system, which places greater emphasis on university degrees as a key pathway to employment compared to Germany’s vocational training model (“Ausbildung”).

Marriage rates were significantly higher among Turkish respondents, consistent with national figures [47,48]. Conversely, Germany showed higher rates of divorce, widowhood, and separation, which may in part explain lower levels of social participation [49]. When considered alongside the SSPQ-L results, which showed higher social engagement among Turkish participants, these demographic patterns offer important cultural insights. The stronger emphasis on family-oriented living and communal practices in Turkish culture may foster greater day-to-day social involvement. Even among Turkish migrants in Europe (e.g., in Germany), a prior study has documented active efforts to re-establish social roles through participation in associations, social clubs, and community events [50]. Nonetheless, due to the lack of comparative or nationally representative data on social participation in both countries, this interpretation should be treated with caution.

Employment patterns also followed national trends, with Türkiye exhibiting higher unemployment rates (8.8%) compared to Germany (3.3%) as of November 2024 [51,52]. For context, the OECD average unemployment rate was 4.9% in October 2024 [53]. Germany also had a higher proportion of retirees, in line with its older population structure [52,54,55].

Smoking behaviors also differed significantly. Turkish participants reported a significantly higher prevalence of daily shisha use, whereas non-smoking was more common in Germany. This disparity may stem from differences in public health policies, awareness efforts and anti-smoking campaigns. According to recent WHO reports, daily smoking rates are higher in Türkiye (26.2%) than in Germany (17.2%) [56].

### 4.2. Adherence to the MedDiet and Eating Habits

Adherence to the MedDiet did not differ in total MedLife scores between countries, but notable contrasts emerged across blocks and food items. Turkish participants showed stronger adherence to Mediterranean food components (e.g., legumes, olive oil, nuts), while Germans aligned more with certain recommendations, such as moderate red meat, fish, and cereal intake. These findings support the notion that adherence patterns differ not only by region but also by specific dietary components. The stronger alignment of Turkish respondents with traditional MedDiet food items is consistent with literature showing residual adherence to traditional dietary patterns in Mediterranean countries [28].

In dietary habits (Block 2), Turkish respondents reported greater adherence to guidelines on salt intake, grain products, snacks, and processed foods. While the reported lower salt consumption contradicts national trends showing that Türkiye exceeds the WHO-recommended salt intake of 5g/day [57], the result may reflect growing public awareness and recent national initiatives to reduce salt intake. Regarding processed food consumption, prior research estimates that UPFs contribute approximately 27–30% of daily energy intake in Türkiye, compared to nearly 48% in Germany [58]. This aligns with our finding that Germans more frequently consumed sugary beverages and wine. Per capita wine consumption is significantly higher in Germany (19 L/year) compared to Türkiye (3.2 L/year), largely due to cultural and religious factors [59,60]. Similarly, the German soft drink market is substantially larger, supporting our data on higher sugary drink consumption in Germany [61,62].

In lifestyle behaviors (Block 3), Germans reported higher adherence scores than Turkish participants. These findings reflect stronger alignment among German respondents with behaviors such as meeting physical activity guidelines (as further corroborated by the IPAQ-SF data, discussed in a dedicated section below), regular napping, and achieving the recommended 6–8 h of sleep per night. Conversely, Turkish participants were more likely to limit TV time to less than one hour per day, a behavior associated with reduced sedentary behavior and healthier lifestyle choices, as highlighted in recent research linking screen time to lifestyle patterns and chronic disease risk [63]. Notably, Ammar et al. [64] demonstrated that prolonged sedentary behaviors, such as screen-based sitting, are significantly associated with a higher burden of cardiometabolic and neurodegenerative diseases across diverse populations. These results suggest differing lifestyle priorities shaped by social norms and work–life balance structures. When summing scores across all three blocks, the total MedLife Index score did not significantly differ between Germany and Türkiye, with the majority of participants in both countries classified as medium adherents. A 2022 systematic review highlighted that traditional MedDiet patterns are steadily declining in many Mediterranean countries, while non-Mediterranean nations like Germany are increasingly promoting and adopting MedDiet principles through public health efforts [29]. Accordingly, our findings suggest that despite Türkiye’s geographic and cultural proximity to the Mediterranean, this does not automatically translate to stronger adherence. In fact, Germany (a non-Mediterranean country) exhibited greater alignment with several MedDiet recommendations (e.g., cereal and moderate wine consumption) and appears to be integrating these principles more effectively into everyday practices, likely facilitated by structured health promotion strategies. Nevertheless, despite higher awareness and accessibility, secular dietary habits, convenience-oriented food culture, and reduced home cooking frequency may limit full adherence.

Conversely, adherence patterns in Türkiye are influenced by cultural and religious norms, including halal food and beverage preferences, cooking traditions, and the centrality of shared family meals. While restrictions on pork align with MedDiet principles, complete abstinence from alcohol among Muslims leads to lower scores in the “moderate wine consumption” item of the MedLife Index, despite this abstinence reflecting a culturally appropriate health behavior. This highlights the importance of considering culturally sensitive adaptations of the MedLife Index, such as redefining this item as “limited alcohol consumption” (e.g., ≤2 rather than 1–2 servings per day), to enhance inclusivity across populations where alcohol consumption is restricted for religious or ethical reasons. Moreover, economic constraints, urbanization, and the growing availability of Western-style processed foods may further offset potential benefits in Türkiye.

Collectively, these findings underscore that MedDiet adherence is multidimensional and culturally embedded, shaped not only by geography but also by socioreligious context, public health infrastructure, and food environment accessibility [10].

### 4.3. Physical Activity, Social Participation, and Their Association with MedLife Adherence

Physical activity levels were higher in Germany than in Türkiye, consistent with Eurostat data, which indicates that 26.3% of German adults meet WHO’s physical activity recommendations, compared to only 3.2% in Türkiye [65,66]. Several contextual and infrastructural factors may explain this disparity. Germany’s urban environments are generally more conducive to active transportation, offering pedestrian- and cyclist-friendly infrastructure and well-integrated public spaces that encourage movement and recreational activities [67]. In contrast, long commuting times and less supportive infrastructure in many Turkish cities may limit opportunities for physical activity [68,69,70].

Levels of social participation were higher in Türkiye, especially in the “Often socially active” category. This observation reflects Türkiye’s collectivist cultural orientation, where close-knit family ties and community involvement are integral to daily life [71,72]. On the other hand, Germany’s more individualistic and personalistic culture may contribute to the lower levels of social engagement. This is in line with the present sociocultural differences trends, indicating higher rates of marriage and communal living in Türkiye compared to greater proportions of single, divorced, or widowed individuals in Germany, factors which may influence daily social interactions and connectedness.

Correlation analyses provided additional insight into how these lifestyle components interrelate, revealing important cultural distinctions. Among German participants, total MedLife Index scores showed a moderate positive association with social participation and a weaker but significant association with physical activity. These findings suggest that German participants, who adhere more closely to the MedLife, are also more likely to be socially engaged and physically active, though the strength of these associations differs. The moderate association with social participation further highlights the relevance of conviviality, group-based meals, and social cohesion as fundamental elements of MedLife, as emphasized by Bach-Faig et al. [7] and Ammar et al. [10]. The positive association with physical activity aligns with recent evidence from multiple linear regression models conducted by Ammar et al. [10], which demonstrated that physical activity significantly contributes to MedLife adherence, while prolonged sitting time is negatively associated with it. These results are also consistent with previous findings by García-Hermoso et al. [73], who reported that individuals adhering to the MedDiet are more likely to engage in regular physical activity. Similarly, studies by Mendes et al. [74] and Júdice et al. [75] observed an inverse relationship between sedentary behavior and adherence to the MedLife.

Among Turkish participants, a weaker yet significant association was found between MedLife adherence and social participation, while no significant relationship was observed with physical activity. This pattern may reflect a more compartmentalized or fragmented adoption of MedLife elements, in which diet, social engagement, and physical activity are perceived and enacted as separate domains. Several factors may contribute to this segmentation, including regional disparities, socioeconomic differences, and limitations in urban planning or access to structured physical activity programs.

The association between social engagement and adherence to MedDiet and its lifestyle is well supported by existing literature. For instance, findings from the Hellenic Longitudinal Investigation of Aging and Diet (HELIAD) indicated that older adults with higher MedDiet adherence were more frequently involved in social, intellectual, and physical activities [27]. Similarly, Ammar et al. [10] demonstrated through various regression models that social participation, alongside physical activity, made strong positive contributions to MedLife adherence. These findings are consistent with earlier work by Psaltopoulou et al. [76], who emphasized the importance of community engagement and active lifestyles in sustaining MedDiet patterns, and Bonaccio et al. [77], who highlighted how socialization promotes adherence by embedding healthy eating within collective and culturally meaningful practices.

Collectively, these findings emphasize the cultural sensitivity and multidimensional nature of MedLife adherence. In this study, German participants exhibited a more integrated adoption of dietary, social, and physical activity components, whereas Turkish participants demonstrated stronger engagement within the social domain but weaker interconnections across lifestyle components. These insights highlight the necessity for culturally tailored, context-specific interventions that move beyond one-size-fits-all approaches. Effective strategies should account for the broader sociocultural and environmental contexts shaping engagement with MedLife. Future longitudinal and mixed-methods research is warranted to explore how these interconnected lifestyle components evolve and interact over time in diverse populations.

### 4.4. MedDiet Awareness and Perceived Barriers

Awareness of the Mediterranean Diet was significantly higher among Germans, reflecting stronger public health communication and nutrition education strategies. In contrast, while elements of the MedDiet are commonly practiced in coastal and rural areas of Türkiye, they may not be conceptualized or labeled explicitly as part of the “Mediterranean Diet”, a factor that could explain the lower self-reported awareness levels. Prior studies have similarly documented low MedDiet awareness among Turkish university students, highlighting a potential gap in formal nutrition education and public messaging around healthy eating patterns [78,79,80].

Perceived barriers to MedDiet adherence differed markedly between countries. Turkish participants more frequently reported obstacles related to price affordability, attitudinal concerns, and individual beliefs. These results likely reflect broader socioeconomic challenges, including high inflation, income disparity, and food insecurity, that limit access to minimally processed foods typical of the MedDiet. This interpretation is further supported by our socioeconomic comparison, which revealed higher unemployment rates among Turkish respondents, potentially constraining food purchasing power. Additionally, Türkiye’s diverse cultural and regional dietary practices, shaped by its position as a bridge between Europe and Asia, may sustain alternative eating norms that compete with MedDiet recommendations [81,82]. Conversely, German participants reported a higher prevalence of barriers related to social norms, lack of time, and insufficient cooking skills or nutritional knowledge. These patterns align with evidence of entrenched reliance on processed and convenience foods and a broader decline in home cooking, as shown in the German Health Interview and Examination Survey (DEGS1, 2008–2011), where only about 50% of adults still prepare fresh hot meals daily [83,84]. Other obstacles, such as medical conditions, food allergies, and personal dietary restrictions, were also more frequent among German participants, indicating that even populations with higher awareness may face lifestyle- and health-related limitations affecting adherence.

Overall, barriers appear structural and economic in Türkiye but behavioral in Germany, underscoring the need for culturally sensitive, context-specific strategies.

### 4.5. Strengths and Limitations

When interpreting the results of this study, it is important to consider both its strengths and limitations. A key strength lies in its cross-cultural scope, comparing two distinct populations, Germany and Türkiye, using a balanced sample size with sufficient statistical power, supporting the robustness and internal validity of the cross-country comparisons. Additionally, the study employed a multicomponent framework and validated questionnaires to comprehensively assess adherence to MedLife, capturing dietary patterns, physical activity, and social behaviors. However, several limitations should be acknowledged. First, the Turkish sample was primarily drawn from Istanbul, while the German sample, although regionally diverse, was recruited through an online convenience approach. Thus, the results are not nationally representative and should be viewed as context-specific, reflecting adults living in urbanized environments with comparable sociodemographic and cultural profiles. Second, cultural differences between Germany and Türkiye could influence how lifestyle behaviors, particularly those assessed by the MedLife index, are perceived and reported, potentially introducing bias in cross-cultural comparisons. Third, the current study focused exclusively on data from Germany and Türkiye, despite the broader multinational scope of the MEDIET4ALL survey. This choice was based on the completeness, balance, and quality of the available data from these two countries at the time of analysis. Nevertheless, this selective inclusion may introduce a degree of selection bias and limit generalizability to the broader Mediterranean region. Fourth, the use of an online self-reported survey introduces potential biases, including social desirability bias, recall bias, and misinterpretation of questions. In particular, both BMI and self-rated health status were based on self-reported data, which may compromise accuracy due to variability in individual perception and the tendency to under- or over-report weight or health conditions. These factors may affect the reliability of health classification and introduce response bias. Additionally, the cross-sectional design limits the ability to establish causal relationships, as it captures associations at a single point in time without the capacity to track behavioral changes or health outcomes over time. Taken together, these methodological constraints highlight the need for cautious interpretation of the findings.

Future research should adopt longitudinal and mixed-method designs to track behavioral changes over time, thereby addressing current limitations related to causality and temporal dynamics. The use of more precise and/or objective measurement tools and monitoring methods would enhance the accuracy of findings and provide stronger evidence for causal relationships between MedLife adherence and MedDiet-related behaviors. Developing country-specific strategies to promote MedLife is also essential for understanding and addressing cross-country differences. Comparative studies involving a broader range of Mediterranean and neighboring countries, beyond the current focus on Germany and Türkiye, are also strongly recommended to enrich cross-cultural understanding, enhance policy relevance, and allow better evaluation of regional variation in MedLife adherence. Furthermore, studies involving larger and more regionally balanced samples will improve the reliability and generalizability of findings. To build on the insights gained from this study, future research should incorporate these methodological improvements, including leveraging the full multinational MEDIET4ALL dataset, to strengthen the evidence base.

### 4.6. Practical Applications

The findings of this study offer valuable insights for designing culturally tailored strategies to promote adherence to MedLife in Germany and Türkiye. Country-specific public health interventions are essential to address distinct patterns of behavior and perceived barriers. In Türkiye, efforts should prioritize addressing economic constraints, as participants reported higher barriers related to price affordability, attitudinal concerns, and individual beliefs. Given the higher unemployment rate and broader economic challenges, policies that support food subsidies, affordable pricing of minimally processed foods, and targeted nutritional support for low-income populations are crucial. Public campaigns should also aim to raise awareness about the MedDiet as a formal health-promoting concept, especially among urban populations, while respecting diverse regional food practices. In Germany, where participants reported greater challenges related to social norms, lack of time, cooking skills, and medical or dietary restrictions, interventions should focus on increasing awareness, culinary literacy, and integration of MedDiet principles into traditional German meals. Cooking workshops, recipe adaptations, and supermarket campaigns featuring Mediterranean foods may help shift public perceptions and increase accessibility.

Across both countries, educational programs in schools, workplaces, and healthcare settings should promote knowledge of the MedLife, emphasizing not only healthy food choices but also behaviors linked to physical activity, rest, and social interaction, as highlighted in Block 3 of the MedLife Index. In Germany, enhancing urban planning to support active commuting and community-based physical activity programs could reinforce these behaviors. In Türkiye, leveraging the strong social fabric to organize communal meals, neighborhood activities, and support groups may further enhance adherence, particularly in light of higher reported social participation.

Further innovation in digital health solutions, such as mobile applications and interactive platforms, should focus on features like personalized meal planning, meal preparation videos/steps, activity tracking, and motivational prompts to support adherence to the MedLife. These tools could be particularly effective for individuals with limited cooking skills or nutritional knowledge, and for younger, tech-savvy populations, by offering accessible and engaging support in diverse daily life contexts.

Finally, policymakers, educators, and healthcare providers should collaborate with the food industry to promote the availability of affordable Mediterranean food options, reformulate processed foods to align with MedDiet principles, and reduce barriers to adoption. Through coordinated, culturally sensitive, and multi-level strategies, stakeholders can foster long-term adherence to the MedLife and contribute to the prevention of non-communicable diseases in both Mediterranean and non-Mediterranean countries.

## 5. Conclusions

This study highlights important cross-cultural variations in lifestyle behaviors associated with MedLife adherence, shaped by diverse social, cultural, and economic contexts in Germany and Türkiye. While both countries demonstrated moderate overall adherence to the MedDiet, notable differences emerged at the subcomponent level. Turkish participants showed higher adherence to core Mediterranean food items (e.g., legumes and olive oil), whereas German participants reported greater engagement in lifestyle-related behaviors such as meeting sleep and physical activity recommendations. Awareness of the MedDiet was significantly higher in Germany; however, perceived barriers varied substantially: Turkish respondents more frequently cited affordability and attitudinal beliefs, whereas German participants reported greater challenges related to social norms, cooking skills, and personal health conditions.

A key insight of this study is the moderate positive association between MedLife adherence and social participation, particularly in the German sample, underscoring the importance of conviviality and social interaction in sustaining healthy lifestyle behaviors. However, the relatively weak correlation between MedLife adherence and physical activity suggests the need for more integrative strategies that bridge dietary and movement-related behaviors. These findings call for context-specific public health strategies. In Türkiye, interventions should focus on improving affordability and raising awareness through culturally relevant education and policy support. In Germany, efforts might prioritize promoting the cultural acceptance of Mediterranean eating habits, enhancing cooking literacy, and addressing practical barriers to dietary change.

Given the cross-sectional design and the representative sampling limitations, particularly in Türkiye, these findings should be interpreted with caution and further validated through larger, longitudinal, and mixed-methods research. The present results should be viewed as context-specific to the study populations and not directly generalized to the wider Mediterranean or European populations. Future studies are encouraged to explore how dietary, physical, and social lifestyle dimensions interact over time and how targeted interventions can be effectively adapted to different cultural environments.

## Figures and Tables

**Table 1:** Sociodemographic characteristics of the study population (n = 4010) stratified by countries (Germany vs. Türkiye).

	Total	Germany	Türkiye	*p*-Value
Living Environment, n (%)				<0.001
Urban environment	850 (71.8)	343 (56.3)	507 (88.2)	
Suburban environment	169 (14.3)	132 (21.7)	37 (6.4)	
Rural environment	165 (13.9)	134 (22.0)	31 (5.4)	
Sex, n (%)				0.675
Male	560 (47.5)	284 (46.9)	276 (48.1)	
Female	620 (52.5)	322 (53.1)	298 (51.9)	
Age Category, n (%)				<0.001
18–35 years	663 (56.0)	302 (49.6)	361 (62.8)	
36–55 years	372 (31.4)	178 (29.2)	194 (33.7)	
55+ years	149 (12.6)	129 (21.2)	20 (3.5)	
BMI Category, n (%)				0.046
Underweight	44 (3.7)	14 (2.3)	30 (5.2)	
Normal Weight	608 (51.4)	323 (53.0)	285 (49.6)	
Overweight	371 (31.3)	186 (30.5)	185 (32.2)	
Obesity	161 (13.6)	86 (14.1)	75 (13.0)	
Education, n (%)				<0.001
No schooling completed	81 (6.8)	9 (1.5)	72 (12.5)	
High school graduation	296 (25.0)	163 (26.8)	133 (23.1)	
Professional degree	193 (16.3)	170 (27.9)	23 (4.0)	
Bachelor’s degree	436 (36.8)	151 (24.8)	285 (49.6)	
Master’s degree/doctorate	178 (15.0)	116 (19.0)	62 (10.8)	
Marital Status, n (%)				<0.001
Single	570 (48.1)	320 (52.5)	250 (43.5)	
Married living as a couple	525 (44.3)	221 (36.3)	304 (52.9)	
Widowed, divorced, separated	89 (7.5)	68 (11.2)	21 (3.7)	
Employment Status, n (%)				<0.001
Employed	772 (67.0)	397 (66.3)	375 (67.8)	
Unemployed	82 (7.1)	22 (3.7)	60 (10.8)	
Student	232 (20.1)	126 (21.0)	106 (19.2)	
Retired	66 (5.7)	54 (9.0)	12 (2.2)	
Health Status, n (%)				0.291
Healthy	896 (79.4)	450 (79.6)	446 (79.2)	
At risk	152 (13.5)	81 (14.3)	71 (12.6)	
With diseases	80 (7.1)	34 (6.0)	46 (8.2)	
Smoking Habits, n (%)				<0.001
Yes (cigarettes)	344 (29.1)	152 (25.0)	192 (33.4)	
Yes (shisha)	130 (11.0)	28 (4.6)	102 (17.7)	
No	710 (60.0)	429 (70.4)	281 (48.9)	

**Table 2:** Analysis of the MedLife Index: Mediterranean diet adherence, dietary habits, and consumption patterns by countries (Germany vs. Türkiye).

	Criteria for 1 Point	Total	Germany	Türkiye	*p*
Block 1: Mediterranean Food Consumption, n (%) for 1 point					
Sweets	≤2 servings/week	663 (56.0)	328 (53.9)	335 (58.3)	0.127
Red meat	<2 servings/week	639 (54.0)	351 (57.6)	288 (50.1)	0.009
Processed meat	≤1 serving/week	727 (61.4)	301 (49.4)	426 (74.1)	<0.001
Eggs	2–4 servings/week	424 (35.8)	249 (40.9)	175 (30.4)	<0.001
Legumes	≥2 servings/week	684 (57.8)	285 (46.8)	399 (69.4)	<0.001
White meat	2 servings/week	265 (22.4)	108 (17.7)	157 (27.3)	<0.001
Fish	≥2 servings/week	220 (18.6)	128 (21.0)	92 (16.0)	0.027
Potatoes	≤3 servings/week	1000 (84.5)	511 (83.9)	489 (85.0)	0.590
Dairy	2 servings/d	170 (14.4)	75 (12.3)	95 (16.5)	0.039
Nuts/Olives	1–2 servings/d	710 (60.0)	334 (54.8)	376 (65.4)	<0.001
Herbs/Spices	≥1 serving/d	1063 (89.8)	548 (90.0)	515 (89.6)	0.812
Fruits	3–6 servings/d	166 (14.0)	97 (15.9)	69 (12.0)	0.052
Vegetables	≥2 servings/d	503 (42.5)	265 (43.5)	238 (41.4)	0.460
Olive oil	≥3 servings/d	271 (22.9)	104 (17.1)	167 (29.0)	<0.001
Cereals	3–6 servings/d	329 (27.8)	201 (33.0)	128 (22.3)	<0.001
Block 1: Total Score			6.38 ± 1.98	6.87 ± 1.73	<0.001
Block 2: Mediterranean Dietary Habits, n (%) for 1 point					
Drink	6–8 servings/d or ≥3 servings/week	988 (83.4)	505 (82.9)	483 (84.0)	0.618
Wine	1–2 servings/d	54 (4.6)	43 (7.1)	11 (1.9)	<0.001
Salt	Limit salt: Yes	759 (64.1)	358 (58.8)	401 (69.7)	<0.001
Grain products	Yes/fiber > 25 g/d	743 (62.8)	363 (59.6)	380 (66.1)	0.021
Snacks	≤2 servings/week	783 (66.1)	386 (63.4)	397 (69.0)	0.040
Nibbling	Limit nibbling between meals: Yes	721 (60.9)	387 (63.5)	334 (58.1)	0.054
Sugar	Limit sugar in beverages: Yes	851 (71.9)	472 (77.5)	379 (65.9)	<0.001
Block 2: Total Score			4.13 ± 1.68	4.15 ± 1.57	0.835
Block 3: Physical activity, rest, Social Habits, and Conviviality, n (%) for 1 point					
Physical activity	>150 min/week or 30 min/d	789 (66.6)	423 (69.5)	366 (63.7)	0.034
Nap	Yes	352 (29.7)	204 (33.5)	148 (25.7)	0.004
Sleep	6–8 h/d	884 (74.7)	477 (78.3)	407 (70.8)	0.003
Watching TV	<1 h/d	223 (18.8)	93 (15.3)	130 (22.6)	0.001
Time with friends	≥2 h/weekend	872 (73.6)	458 (75.2)	414 (72.0)	0.211
Team sports	≥2 h/week	319 (26.9)	163 (26.8)	156 (27.1)	0.887
Block 3: Total Score			2.99 ± 1.27	2.82 ± 1.30	0.027
MedLife Index Categories					
Low		391 (33.0)	220 (36.1)	171 (29.7) *	0.005
Medium		589 (49.7)	275 (45.2)	314 (54.6) *	
High		204 (17.2)	114 (18.7)	90 (15.7) *	

*: significant difference compared to Germany at *p* < 0.05.

**Table 3:** Country differences (Germany vs. Türkiye) in MedDiet awareness and potential barriers (MBQ) influencing their adherence to MedLife.

		Total	Germany	Türkiye	*p* Value
MedDiet awareness, n (%)		571	342 (55.5)	229 (38.4)	<0.001
MBQ	Attitudes (suitability, taste, restrictive, food waste)	1000	495 (80.4)	505 (84.7)	0.045
	Social norms (food culture)	996	546 (88.6)	450 (75.5)	<0.001
	Low motivation	209	117 (19)	92 (15.4)	0.101
	Price affordability	915	422 (68.5)	493 (82.7)	<0.001
	Time/effort for consuming	304	198 (32.1)	106 (17.8)	<0.001
	Low accessibility/availability of Mediterranean food	1060	532 (86.4)	528 (88.6)	0.242
	Lack of knowledge and cooking skills	207	123 (20)	84 (14.1)	0.007
	Food allergies and intolerances	146	101 (16.4)	45 (7.6)	<0.001
	Cultural and/or religious reason	1212	594 (96.4)	531 (89.1)	<0.001
	Medical reason	1163	569 (92.4)	567 (95.1)	0.047
	Individual beliefs (e.g., vegan and vegetarian)	1108	536 (87)	572 (96)	<0.001
	Taste dislike	251	119 (19.3)	132 (22.1)	0.224
	Other	21	13 (2.1)	8 (1.3)	0.306
MBQ total score			7.09 ± 1.24	6.9 ± 1.21	0.16

**Table 4:** IPAQ-SF and SSPQ-L Scoring Categories by Country of Living (Germany vs. Türkiye).

	Total	Germany	Türkiye	*p*-Value
Participants’ categories according to the IPAQ-SF score, n (%)				<0.001
Low Activities	675 (57.0)	271 (44.5)	404 (70.3)	
Moderate Activities	218 (18.4)	141 (23.2)	77 (13.4)	
High Activities	291 (24.6)	197 (32.3)	94 (16.3)	
Participants’ categories according to the SSPQ-L score, n (%)				0.006
Never	13 (1.1)	8 (1.3)	5 (0.9)	
Rarely	233 (20.2)	137 (22.9)	96 (17.3)	
Sometimes	574 (49.7)	306 (51.2)	268 (48.2)	
Often	290 (25.1)	125 (20.9)	165 (29.7)	
All Times	44 (3.8)	22 (3.7)	22 (4.0)	

**Table 5:** Pearson correlation between MedLife, IPAQ-SF, and SSPQ-L scores among German and Turkish participants.

Variables	MedLife	IPAQ-SF	SSPQ-L
German responders			
MedLife	-		
IPAQ-SF	0.092 *	-	
SSPQ-L	0.349 **	0.159 **	-
Turkish responders			
MedLife	-		
IPAQ-SF	0.071	-	
SSPQ-L	0.093 *	0.178 **	-

* *p* < 0.05, ** *p* < 0.001.

## Data Availability

The datasets generated and analyzed during the current study are not publicly available at this time as further analyses are ongoing, and additional publications based on these data are in preparation. Data may be made available upon reasonable request to the corresponding author once all planned analyses and publications are completed.
